# Special Issue “Purinergic Signalling in Physiology and Pathophysiology 2.0”

**DOI:** 10.3390/ijms27020852

**Published:** 2026-01-15

**Authors:** Ronald Sluyter

**Affiliations:** Molecular Horizons and School of Science, University of Wollongong, Wollongong, NSW 2522, Australia; rsluyter@uow.edu.au

Despite its unpopular beginnings [1], purinergic signalling has emerged as a well-established communication network between cells, tissues, and organs. This network comprises an array of transmembrane adenosine (P1) and nucleotide (P2X and P2Y) receptors, transmembrane and soluble enzymes, nucleoside and nucleotide release and uptake pathways, and accessory molecules [2]. Purinergic signalling plays central and diverse roles in physiology and pathophysiology in humans [3] and other species [4]. Further research, however, is required to improve our understanding of this network and its applications in health and disease in humans and other species. This Special Issue of *International Journal of Molecular Sciences*, “Purinergic Signalling in Physiology and Pathophysiology 2.0” (https://www.mdpi.com/journal/ijms/special_issues/7MG23NJWRF, accessed on 12 November 2025), continues this pursuit, expanding on the inaugural Special Issue on this topic [5]. This Editorial introduces this second Special Issue, comprising two reviews and seven original research articles.

The review by Mata-Martínez et al. covers purinergic signalling in non-parenchymal liver cells, providing an excellent example of the diversity of purinergic molecules and functions within a given organ. This article begins with a brief overview of chronic liver diseases, introducing the reader to inflammation, fibrosis, cirrhosis, and hepatocellular carcinoma. After introducing purinergic signalling more broadly, the article explores the ligands, P1 and P2 receptors, and ectonucleotidases involved in purinergic signalling in hepatic stellate cells, Kupffer cells, liver sinusoidal endothelial cells, and biliary epithelial cells (cholangiocytes). Sections for each cell type are supported by tables providing valuable summaries of key observations. The article concludes by discussing past and current studies to therapeutically target purinergic signalling pathways in non-parenchymal liver cells.

The review by Cheah et al. provides a timely overview of P2X7 and its genetic variants. Based on cryo-electron microscopy studies and other research, this article provides an up-to-date overview of the structure of P2X7 and its gating mechanisms and binding sites for adenosine 5′-triphosphate (ATP) and negative and positive allosteric modulators. The authors then give a comprehensive summary of single-nucleotide polymorphisms (SNPs) within the human *P2RX7* gene and their relationship with P2X7 expression and function and disease. Known haplotypes of the human *P2RX7* gene and predictions regarding their impact on receptor function and disease associations are outlined. The various splice variants (isoforms) of human P2X7 and their potential impact when assemble as heterotrimers are also presented. The article calls for greater consideration of these SNPs, haplotypes, and isoforms in structure/function studies of P2X7, as well as in interpreting past clinical trials and designing future ones with P2X7 antagonists or when considering P2X7 as a prognostic or diagnostic biomarker. Finally, the article outlines the potential of in silico studies to better understand the structures and functions of P2X7 and its variants.

Three original research articles focus on P2 receptors. Salcman et al. characterised P2X7 in human mast cells derived from cultures of CD117^+^ blood progenitors. This article shows that ATP but not adenosine 5′-diphosphate (ADP) can induce mast cell degranulation and that this process is enhanced by priming with interleukin (IL)-33 but not by another alarmin, thymic stromal lymphopoietin (TSLP). Although IL-33 increased P2X1, P2X4 and P2X7 on these cells, the observed effects of ATP were largely mediated by P2X7 activation, with additional contributions from P2X1 or P2X4 when activated with P2X7. IL-33 priming also promoted ATP-induced IL-8 release from human mast cells, as well as ATP-induced ERK1/2 signalling in these cells. Collectively, this study provides further insights into the contribution of purinergic signalling in mast cells and inflammation.

Niyadurupola et al. explored P2X7 activation in human retinal ganglion cells from organotypic retinal cultures. This article demonstrates that the potent P2X7 agonist 2′(3′)-*O*-(4-benzoylbenzoyl)ATP (BzATP) induces IL-1β and IL-10 release from these cells. The former cytokine was then studied more thoroughly. P2X7 activation induced both IL-1β mRNA expression and protein release. Notably, exogenous IL-1β protected retinal ganglion cells from P2X7-mediated cell death via activation of the type-1 IL-1 receptor. This effect of IL-1β was contrary to expectations, with prior studies reporting a neurotoxic role of this cytokine in these cells from rodents [6,7]. It remains unknown why P2X7 activation induced death in retinal ganglion cells despite inducing IL-1β release. Thus, the data suggest a neurodegenerative and neuroprotective role for the ATP/P2X7/IL-1β axis in the human retina, but further studies are required to understand purinergic signalling in retinal diseases such as glaucoma.

Sophocleous et al. compared P2Y_1_ and P2Y_12_ on human, dog, and cat platelets. Immunoblotting indicated the presence of both receptors in the platelets from each species. After establishing a modified 384-well plate aggregation assay, this article showed that the P2Y_1_ and P2Y_12_ agonists ADP and 2-methylthio-ADP induce the aggregation of platelets from all three species. This aggregation was mainly mediated via P2Y_12_ activation, with a minor contribution via P2Y_1_ activation. Although some species-specific differences were observed in relation to spontaneous and nucleotide-induced platelet aggregation, this study illustrates the potential of platelet P2Y receptors as therapeutic targets to treat thrombosis and other conditions in companion animals and reinforces the contribution of purinergic signalling to platelet function in multiple species.

Three original research articles focus on ectonucleotidases. Using an ex vivo mouse detrusor-free bladder model, Gutierrez Cruz et al. studied the effect of prostaglandins on soluble nucleotidase (sNTD) release from the lamina propria in nondistended and extended bladder settings. sNTD release (measured by ATP hydrolysis) via prostaglandin (PG) receptor activation varied between settings. Endogenous PGE_2_ caused spontaneous but not distention-induced release of sNTDs, an effect mediated via EP2 and EP3 receptors. Exogenous PGE_2_ increased spontaneous sNTD release via EP3, EP4, and LP receptors but increased distention-induced sNTD release via EP1-4 and FP receptors. Exogenous PGD_2_ increased spontaneous sNTD release via DP2 receptors, while exogenous PGD_2_ increased distention-induced sNTD release via DP1 and DP2 receptors. Exogenous PGF2α increased spontaneous but not distention-induced sNTD release via FP receptors. Collectively, this study showed that spontaneous and mechanosensitive release of sNTDs and subsequent ATP hydrolysis are regulated by different PG-mediated pathways. Further, this study showed that both endogenous and exogenous PGs can differentially influence sNTD release from the lamina propria of the bladder. Together, these findings highlight the complexity of PGs and extracellular ATP in the bladder, adding to the important role of purinergic signalling in regulating bladder function.

Using clinical blood samples, Elsaghir et al. investigated the association between the ectonucleotidases CD39 and CD73 and adenosine receptors in hospitalised patients with COVID-19. Proportions of regulatory T cells (Tregs) including CD39^+^ Tregs, as well as CD39^+^CD4^+^ T cells, were increased in patients compared to healthy age- and sex-matched control subjects. Proportions of CD39^+^ Tregs were also greater in those with severe COVID-19 compared to those with moderate COVID-19. None of these cell populations were associated with altered survival. In contrast to findings with CD39, plasma adenosine concentrations were reduced in COVID-19 patients compared to control subjects, with adenosine concentrations lower in severe disease compared to moderate disease. Plasma IL-10 and transforming growth factor-β concentrations showed opposing changes to adenosine in these groups. The expression of mRNA for CD73 in mononuclear cells was decreased in COVID-19 patients compared to control subjects, but this was not the case for adenosine receptors (A_1_, A_2A_, A_2B_, and A_3_). Logistic regression modelling revealed CD39^+^ cell proportions and IL-10 and adenosine concentrations related to disease severity. Collectively, this study provides further evidence for the emerging role of purinergic signalling in COVID-19 disease progression.

In another study using clinical blood samples, Turner et al. investigated the proportions of T and B cells expressing CD39 and CD73 in patients with head and neck squamous cell carcinoma. Combined radiotherapy altered the proportions of CD4^+^ and CD8^+^ T cells and CD19^+^ B cells, including those with or without CD39 and/or CD73 at mid- and post-treatment stages. Proportions of these subsets pre-treatment did not differ between patients with negative or positive human papillomavirus (HPV) status, with the exception of reduced CD73^+^CD4^+^ T cells in the HPV^+^ group compared to the HPV^-^ group. Notably, low pre-treatment CD4^+^:CD8^+^ T cell ratios or CD39^+^CD73^+^CD19^+^ B cell proportions were associated with improved relapse-free survival in patients. This study highlights the prognostic and therapeutic potential of CD39 and CD73 on B cells in head and neck squamous cell carcinoma and provides further support for purinergic signalling in cancer.

The remaining original research article by Matera et al. focuses on the ATP-binding cassette 6 transporter (*ABCC6*), which mediates ATP release from hepatocytes [8]. Matera et al. reported that silencing of the *ABCC6* gene in the human hepatocellular carcinoma Hep G2 cell line up-regulates 256 genes and down-regulates 208 genes. Functional enrichment analyses revealed that these gene alterations were associated mainly with mitochondrial matrix, cell surface, and DNA-binding transcription factor binding (Gene Ontology or GO enrichment) and dilated cardiomyopathy, focal adhesion, and proteoglycans in cancer (Kyoto Encyclopedia of Genes and Genomes or KEGG enrichment). *ABCC6* gene silencing in Hep G2 cells increased adhesion and reduced clonogenic potential and migration. This silencing altered expression of several genes or proteins associated with cell-to-cell and cell-to-matrix interactions or epithelial–mesenchymal transition supporting the initial transcriptomic analyses. This study highlights the potential role of *ABCC6* and ATP release in hepatocellular carcinoma and supports the theory of a varied role for purinergic signalling in cancer.

Collectively, this Special Issue highlights the central and diverse roles of purinergic signalling in physiology and pathophysiology. It is hoped that this Special Issue and the previous one on this topic will continue to promote the importance of this communication network in health and disease and help stimulate future research in this field.
